# Effect of integrating postpartum family planning into the health extension program in Ethiopia on postpartum adoption of modern contraception

**DOI:** 10.29392/001c.13511

**Published:** 2020-08-02

**Authors:** Deborah Sitrin, Gebi H Jima, Anne Pfitzer, China Wondimu, Tigist Worku Belete, Tsigue Pleah, Berhane Assefa, Tenaye Kebede, Efrem Regassa, Ezedin Aman Usman, Saifuddin Ahmed

**Affiliations:** 1 USAID’s Maternal and Child Survival Program/Jhpiego, Washington, District of Columbia, USA; 2 Arsi University, Assela, Ethiopia; 3 USAID’s Maternal and Child Survival Program/Jhpiego, Addis Ababa, Ethiopia; 4 Jhpiego, Conakry, Guinea; 5 Ministry of Health, Addis Ababa, Ethiopia; 6 Oromia Regional Health Bureau, Ministry of Health, Addis Ababa, Ethiopia; 7 Department of Population Family and Reproductive Health, Johns Hopkins Bloomberg School of Public Health, Baltimore, Maryland, USA

**Keywords:** family planning, postpartum, community health workers

## Abstract

**Background:**

Ethiopia has large unmet need for contraception among postpartum women. Community-level services may improve postpartum contraceptive use in Ethiopia and other contexts where home childbirth is common. This study estimated the additional effect of systematically integrating messages on postpartum family planning (PPFP) into community contacts with pregnant and postpartum women, on top of integrated facility contacts.

**Methods:**

The quasi-experimental study was conducted in two districts in Oromia Regional State and used a controlled trial design with random assignment of primary health care units—a health center and surrounding health posts—to intervention and comparison arms. We used the log-rank test and fitted a multivariate Cox proportional hazards regression model to estimate the adjusted hazard ratio (adjHR) and 95% confidence interval (95% CI) for differences in contraceptive uptake by arm. Time from delivery to uptake of modern contraception was the outcome variable. We separately analyzed subsets of women by place of delivery, as this variable interacts with the study arm.

**Results:**

A total of 772 pregnant women were enrolled February–March 2017 and re-interviewed 15 months later (May 2018). Loss to follow-up was 10% in intervention, 7% in comparison areas. Among women who delivered at home, there was higher PPFP adoption by one year postpartum in the intervention arm (35.2%, 95% CI: 28.8–42.4%) versus comparison arm (27.8%, 95% CI: 22.2–34.4%). In the adjusted Cox regression model, women who delivered at home in the intervention arm were 45% more likely to adopt contraception (adjHR1.45, CI: 1.01–2.07). There was no difference by arm for women who delivered in a facility.

**Conclusions:**

Integrating PPFP into community-level services for pregnant and postpartum women and infants may have additional benefit on top of PPFP services at facilities. The intervention benefited women who delivered at home, an important target population in countries like Ethiopia, where many women do not deliver in a facility. This study, implemented under real world conditions, informs the PPFP body of evidence and fills a gap in research on the contribution of community-based PPFP in contexts where services are integrated within maternal, newborn, and child health care in facilities.

The large gap between the number of postpartum women desiring to prevent or delay another pregnancy and the number using modern contraception in Ethiopia^1^ contributes to the high proportion of births occurring at short intervals with 22% of non-first births occurring less than 24 months after the previous birth.^2^ Short pregnancy intervals contribute to poor maternal outcomes, infant mortality, pre-term and low birth weight newborns, and malnutrition and stunting among children under five.^3-7^ Postpartum family planning (PPFP), or increasing contraception initiation within 12 months following childbirth and continued use until at least two years postpartum, could address this unmet need.^8^

Although PPFP is part of the Ethiopian Ministry of Health’s (MOH) costed implementation plan to increase contraceptive use,^9^ all levels of the health system need to do better in helping postpartum women achieve their reproductive goals. Offering contraception to women immediately after birth in a facility is one proven strategy.^10,11^ Yet in Ethiopia, the majority of women deliver at home and would not benefit from integrating PPFP with facility-based childbirth services.^2^ Community-based family planning interventions can improve contraceptive use,^12^ and may be key to improving PPFP in Ethiopia and other contexts where home delivery is common and many women do not receive facility-based antenatal or postnatal care.^2^

Ethiopia has a well-known health extension program, which brings health services closer to families with 38,000 health extension workers (HEWs) working in over 16,500 health posts and conducting home visits.^13^ HEWs have multiple contacts with pregnant and postpartum women; they provide antenatal and postnatal care, child immunization, and nutrition and growth monitoring.^14^ HEWs also counsel on family planning (including fertility awareness methods like the lactational amenorrhea method) and provide contraception; 27% of modern contraceptive users in Ethiopia obtain their method from an HEW.^2,15^ Initially, HEWs provided short-acting methods only (condoms, pills, injectables); in 2009, HEWs were authorized to insert implants, expanding their role in contraceptive access.^16^ In 2011, community volunteers called the Women Development Army (WDA) were added to the health extension program and support HEWs by promoting health services and sharing health information with communities.^17^

The Maternal and Child Survival Program conducted a study to estimate the additional effect of systematically integrating PPFP messages into contacts HEWs and WDA volunteers have with pregnant and postpartum women, on top of integrated visits at health centers. Study staff worked with the MOH to integrate PPFP counseling, services, and documentation at all primary care health centers in two districts (*woredas*). Sites were then randomized to receive additional inputs to integrate PPFP into HEW and WDA contacts with women or not. This paper compares PPFP adoption in areas with and without PPFP integration into HEW and WDA contacts.

## METHODS

### STUDY SETTING

The study was conducted in the Oromia Region, Ethiopia, in two adjacent *woredas* (districts) with an estimated population of approximately 300,000 people. Oromia Region is the most populous region in Ethiopia and has a modern contraceptive prevalence rate (mCPR) below the national average.^2^ The *woredas* were selected with input from the MOH and were limited to those with sufficient population size for the study and absence of food insecurity, which, based on a pre-study assessment, is associated with diminished contraceptive use.

The selected *woredas* had eight health centers (four each) and 47 health posts (one per *kebele*, the smallest administrative unit of Ethiopia). Health centers provide technical oversight to health posts (staffed by HEWs) and HEWs provide technical oversight to WDA volunteers.

### STUDY DESIGN

This quasi-experimental study used a controlled trial design with random assignment of primary health care units (PHCUs)—a health center and surrounding health posts—to intervention and comparison arms to reduce selection bias. Since multiple health posts report to the same health center, randomizing individual health posts would risk contamination.

### SAMPLE SIZE AND RANDOMIZATION

Sample size was calculated to detect a 10% difference in mCPR between arms with 5% margin of error and 80% statistical power, assuming 1.5 design effect and 20% loss to follow-up. A small design effect was considered sufficient because the population is relatively homogeneous in adjoining *woredas* and all participants are postpartum women, plus it was in line with design effects reported for Oromia Region in the 2011 Ethiopia Demographic and Health Survey report.^18^ Assuming 10% mCPR in comparison areas (based on project data of early PPFP adoption^11^) yielded a sample size of 375 women per arm.

Due to the sample size required, one PHCU was randomly assigned to each arm per *woreda*, resulting in two PHCUs per arm. An estimated 27,172 women of reproductive age lived in selected PHCUs.

### STUDY INPUTS

The study staff engaged *woreda* and zonal (administrative unit above *woreda*) MOH by discussing the study purpose and procedures, holding a half day orientation on informed choice and voluntary contraceptive use, jointly conducting supervision, and reviewing supervision findings. During early implementation, the study team found many WDA volunteers were inactive, so MOH officials expedited efforts to revitalize the program and recruit new WDA volunteers throughout the zone, not just study areas.

Prior to enrolling women, study staff supported implementation of MOH’s strategy to integrate PPFP counseling into maternity care in all health centers in the two *woredas*. Study staff trained 29 antenatal care (ANC), labor and delivery (L&D), postnatal care (PNC), and immunization providers on PPFP counseling with a contraceptive technology update in August 2016 and 32 L&D providers on postpartum intra-uterine device (PPIUD) insertion with a refresher on implant insertion and removal in October 2016 (20 providers received both trainings). Due to staff turnover, an additional 10 providers were trained in November 2017. Immediately after training, health centers were given PPIUD insertion kits and registers to keep in maternity wards. Each health center received post-training follow-up visits (November 2016 for initial training, December 2017 for replacement training) to reinforce providers’ knowledge and competencies.

After assignment of PHCUs to a study arm, study staff and MOH officials conducted supervision visits April 2017–March 2018. Intervention health centers received eight visits, comparison health centers received five or six. During supervision, tally sheets were introduced in ANC, L&D, PNC, and immunization units to document the number of women counseled, provided PPFP, or given intra-facility referral. In addition, wall-mounted dashboards were introduced so health center staff could analyze and discuss service data. Tally sheets and dashboards were introduced in intervention health centers in July 2017 and in comparison health centers in November 2017. Introduction was staggered to assess the utility of the tools under a separate research question, with the assumption that four additional months using tally sheets and dashboards in intervention areas would not alter contraception uptake by one year postpartum.

In intervention areas, 19 HEWs were trained in April 2017 on PPFP counseling with a refresher on implant insertion with the Nexplanon^™^ device (to replace Implanon^™^). HEWs in intervention areas were given modified integrated maternal and child health cards (paper documents used to record ANC, PNC, immunization, and growth monitoring services^19^) with space added to document PPFP counseling, method choice, and method adoption at each contact. The growth monitoring section of the modified card also included questions on return of menses and breastfeeding to prompt HEWs to assess postpartum women’s pregnancy risk. Health centers and health posts in intervention areas were also given modified tetanus toxoid cards for distribution to pregnant women; cards recorded contraceptive method choice, and women were instructed to bring their cards to the facility for childbirth. Intervention health posts received quarterly supervision visits (four total) from study and MOH staff.

HEWs in comparison areas did not receive PPFP training and used standard integrated maternal and child health and tetanus toxoid cards. Study staff conducted no supervision in comparison health posts.

HEWs in intervention areas were tasked with orienting WDA volunteers on PPFP and how to use a pictorial tool to share information on PPFP and track women’s PPFP choices and uptake as well as return of menses, breastfeeding patterns, and return of sexual activity to prompt the WDA to refer women at risk of pregnancy for family planning. See table 1 for study inputs.

### DATA COLLECTION

Women were enrolled in their 2^nd^ and 3^rd^ trimester of pregnancy in February–March 2017. To identify eligible women, data collectors attempted to get lists of pregnant women from health posts. Since records were incomplete, HEWs and *kebele* chairpersons identified local guides from recent health campaigns who would know which households had pregnant women. Data collectors traveled with the guides to households to obtain consent and conduct enrollment interviews to collect demographic information, birth history, contraceptive knowledge, contraceptive use prior to pregnancy, and intention to use contraception after birth. Interviewed women were also asked to refer other pregnant women. The minimum required sample size for the comparison arm was not reached, so additional women were enrolled from six adjoining *kebeles*. Since they were enrolled into the comparison arm, no additional inputs were required to expand the community-level intervention. The two health centers servicing these additional *kebeles* were added to the study as comparison sites and received study supervision visits to ensure the same quality of PPFP services at facility-level. (Health centers had received PPFP training so no additional training was needed).

Women were re-interviewed in May 2018 to collect endline information on contacts with the health system during and after pregnancy, information received on family planning, use of contraception since delivery, and infant feeding and immunization. Contact information collected at enrollment was used to find women; 14 women re-interviewed had moved, most within the same *kebele* except one who moved from an intervention to a comparison area, one from a comparison to an intervention area, and three moved outside the study area. Analysis used arm at time of enrollment.

Questionnaires were developed and translated into two local languages. Data collectors received 5 days of training with mock interviews to improve reliability. Data were collected on tablets using CommCare software (www.dimagi.com/commcare/) with built-in validation checks and data encryption. Field supervisors observed interviews and checked completed surveys; study staff checked data completeness and consistency after data were uploaded from tablets.

During supervision of health centers and intervention health posts, study staff also collected data on human resources, commodity stock-outs, and other health system requirements; observations on quality of care; provider knowledge; client satisfaction; and service statistics extracted from records kept at centers/posts. Comparison health posts did not receive study supervision, but service statistics were extracted from reports sent to health centers.

### DATA ANALYSIS

Data were analyzed using Stata v14. Data quality was checked and cleaned before analysis. Sociodemographic characteristics collected at enrollment were analyzed to examine comparability of arms with differences assessed using Pearson chi-squared test with Rao-Scott correction for categorical variables and Wald test for continuous variables. Household placement within national wealth quintiles was determined by analyzing questions asked at endline using the Equity Tool (equitytool.com), which determines wealth status by comparing to national data.

We calculated the proportion of women in intervention and comparison areas who initiated modern contraception (IUD, implant, injectable, pill, sterilization, condoms, lactational amenorrhea [LAM]) at any time prior to endline interviews. We also reported the type of method and source for the method categorized into HEW, hospital or health center, private provider or drug shop, and other sources. Multiple responses were allowed for both questions since women may have initiated more than one type of contraception in a one-year period. For women who did not initiate modern contraception at any time prior to endline interview, we report the reasons for not adopting contraception (multiple responses allowed).

To explore differences in contraceptive uptake by arm, we used the log-rank test and fitted a multivariate Cox proportional hazards regression model to estimate the adjusted hazard ratio (adjHR) and 95% confidence interval (95% CI). Time from delivery to uptake of modern contraception (IUDs, implants, pills, injectables, condoms, tubal ligation) was the outcome variable. LAM was excluded because it loses effectiveness past six months postpartum. For women interviewed before 12 months postpartum, time was censored at the month of interview if modern contraception not adopted. Time was censored at 12 months for women who adopted modern contraception more than 12 months postpartum. Women pregnant at endline were censored the month they became pregnant; women who had another birth following the index birth were censored before one month postpartum.

Because study arm and place of delivery interact, we constructed a main predictor variable with four categories: comparison arm home delivery, comparison arm facility delivery, intervention arm home delivery, and intervention arm facility delivery. The model also controlled for woman’s age, education, marital status, religion, household wealth status, and number of living children based on factors associated with modern contraceptive use in previous research.^20-24^ The number of living children was estimated using the number reported at enrollment plus the number of babies from the index birth still alive at endline. All analyses were adjusted for clustering at village (*gare*) level for inference. Study participants lived in 269 villages (1–15 per village).

### ETHICAL CONSIDERATION

Ethical approval was obtained from the Institutional Review Boards of the Johns Hopkins Bloomberg School of Public Health and Oromia Regional Health Bureau. The study is registered at ClinicalTrials.gov, NCT03585361.

### ROLE OF THE FUNDING SOURCE

This study was made possible by the generous support of the American people through the United States Agency for International Development (USAID) under the terms of Cooperative Agreement AID-OAA-A-14-00028 and The Bill & Melinda Gates Foundation. Contents are the responsibility of authors and do not necessarily reflect the views of funders or U.S. Government. Funders had no role in study design, data collection, analysis, interpretation, or writing this report.

## RESULTS

Data collectors enrolled 390 pregnant women in intervention areas and 382 in comparison areas. Women in the two arms were similar—average age of 26, mostly married, majority Muslim and the rest mainly Ethiopian Orthodox, nearly half had no education, and nearly all lived in households with farming income (slightly higher in comparison areas) (Table 2). A lower percentage of women in intervention areas lived in households raising livestock (12.8% vs 33.8%). Women in comparison areas were representative of the wealth status in the Oromia Region.^2^ There was greater wealth disparity in intervention areas, with more women in the highest wealth quintile (31.1%) and fewer in middle quintiles.

**Figure 1.**
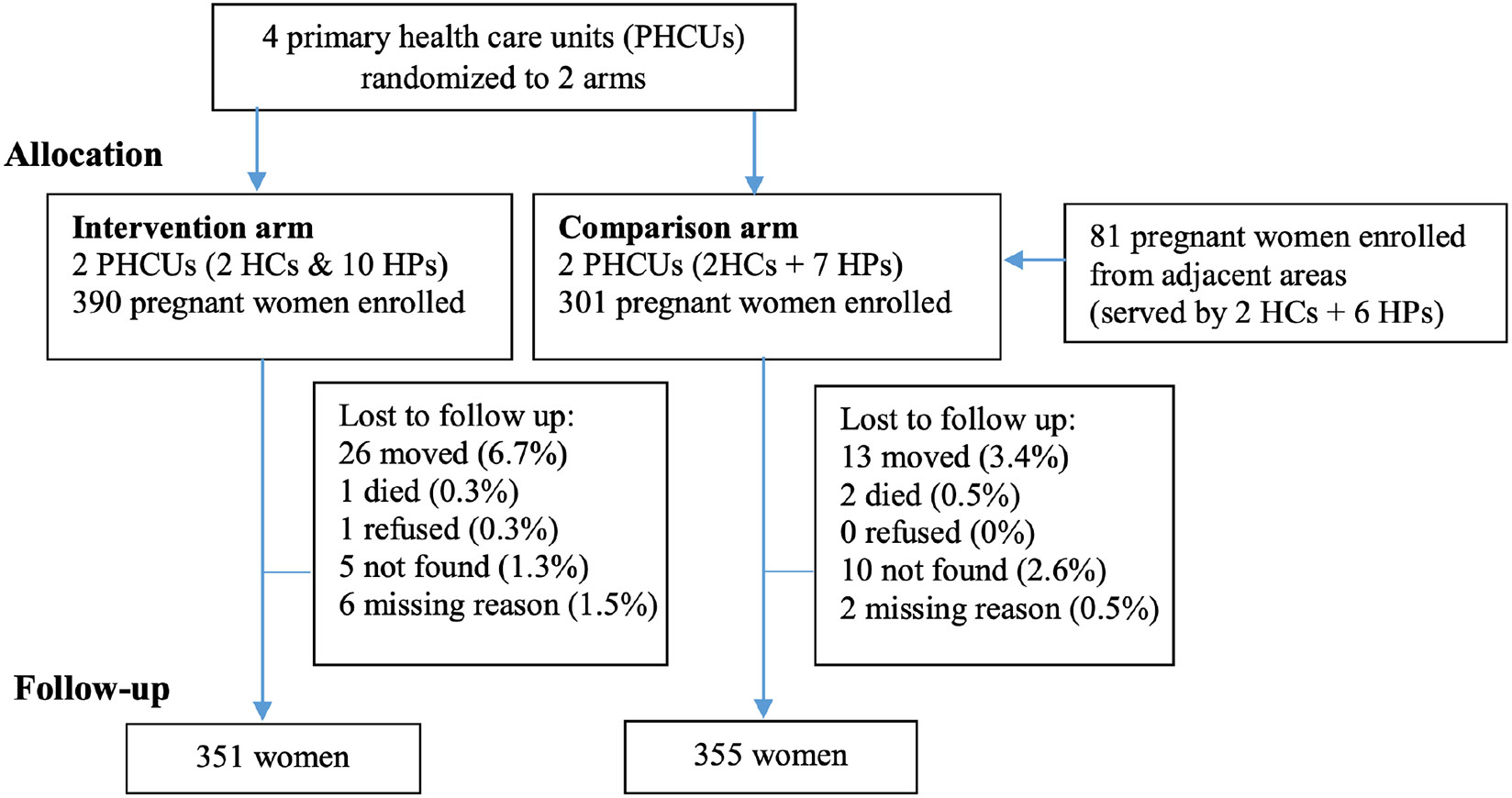
Trial profile: number of participants enrolled during pregnancy and interviewed approximately one year postpartum. HC – Health Centre, HP – Health Post

At endline, 90% of participants in intervention areas and 93% in comparison areas were re-interviewed (Figure 1). Because women were at various stages of pregnancy at enrollment, endline interviews were conducted 10–16 months after the index birth.

**Figure 2.**
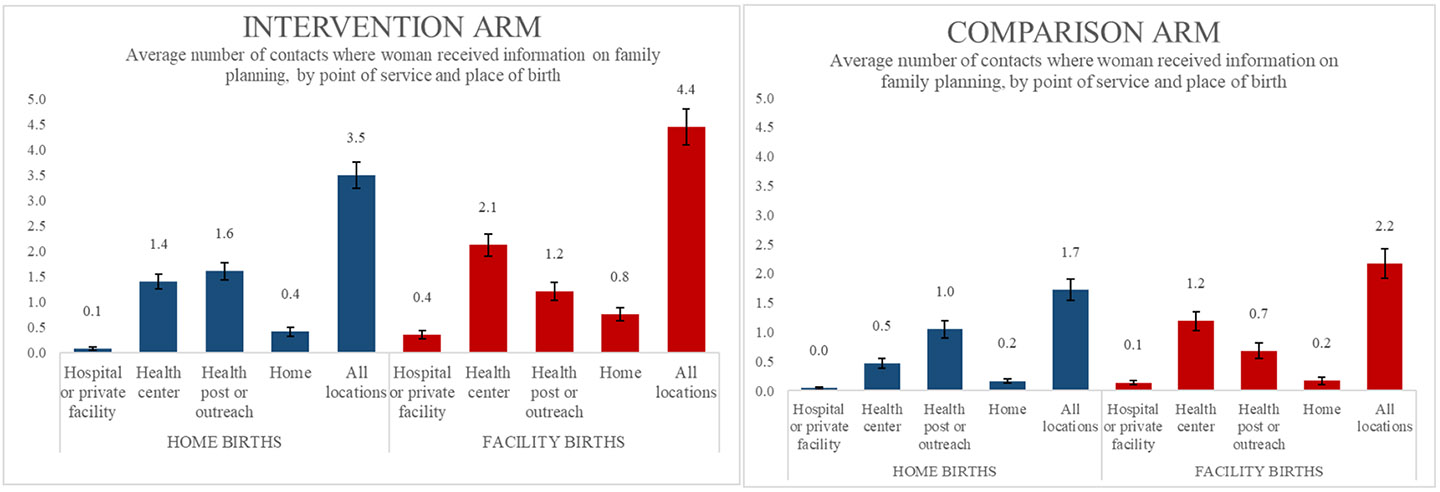
Average number of contacts where woman received information on family planning, by point of service and place of birth.

Figure 2 shows the average number of ANC, delivery, PNC, and child immunization contacts where women reported receiving information on family planning, by point of service, place of delivery, and arm. Women in the intervention arm reported receiving information more frequently from all points of service compared to women in the comparison arm. In both arms, women who delivered in a facility received family planning information most frequently at health centers; women who delivered at home received information most frequently at health posts or via outreach.

Adoption of modern contraception before endline interview was 8.8% higher among women in the intervention arm (48.2% vs 39.94% in comparison). Injectable DMPA was the most common method in both arms. Use of long-acting reversible contraception was higher in the intervention arm, but overall low. The difference was statistically significant for IUDs (1.4% vs 0%, *P*=0.020) but not for implants (9.1% vs 5.6%, *P*=0.101). In both arms, just over one-third of women reported receiving contraception from HEWs. The most common reason for not using contraception among these postpartum women was amenorrhea, followed by pregnancy. Few mentioned other reasons, including opposition from husband or religious objections (Table 3).

**Figure 3.**
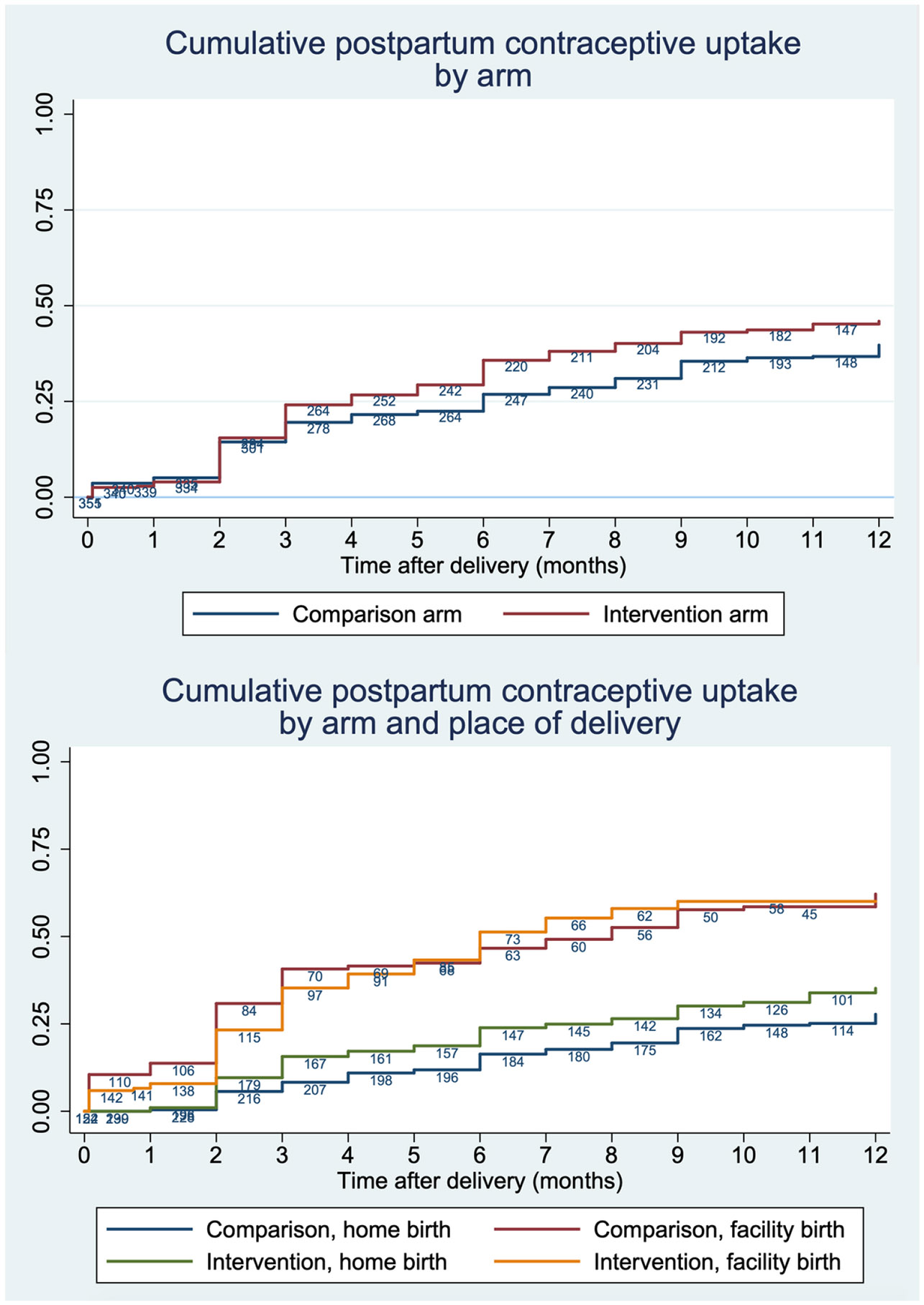
Cumulative postpartum contraceptive uptake by arm and separated by place of delivery.

Women began adopting contraception at higher rates in the intervention arm starting at three months postpartum, but the difference was not statistically significant (log-rank *P*=0.061) (Figure 3). In other words, the rate of uptake did not differ by arm, when place of delivery is not considered. Separating by place of delivery shows higher adoption rates among women who delivered at home in the intervention versus comparison arm. By one year postpartum, 35.2% (95%CI: 28.9–42.4%) of women who delivered at home in the intervention arm and 27.8% (95%CI: 22.2–34.4%) in the comparison arm had adopted modern contraception. Meanwhile, there was little difference between arms among women who delivered in a facility. By one year postpartum, 60.0% (95%CI: 52.3–67.9%) of women who delivered at facility in the intervention arm and 62.2% (95%CI: 53.3–71.1%) in the comparison arm had adopted modern contraception.

Postpartum contraceptive uptake was low the first two months after delivery in both arms, regardless of place of delivery. Low uptake among facility births was confirmed with data extracted during supervision, which also showed low pre-discharge uptake in the initial months of supervision, when most study participants delivered (data not shown).

In the adjusted Cox regression model (Table 4), women who delivered at home in the intervention arm were 45% more likely to adopt contraception over the first year postpartum compared to women who delivered at home in the comparison arm (adjHR1.45, 95%CI: 1.02–2.04). Women who delivered at facility were more likely to adopt contraception over women who delivered at home, with no difference by arm.

## DISCUSSION

Study results suggest that integrating PPFP into community-level services for pregnant and postpartum women and infants may have additional benefit in addition to PPFP integration at facilities, in a context where community workers provide maternal and child health care and family planning services. The intervention benefitted women who delivered at home in that they were 45% more likely to adopt contraception by 12 months postpartum than women in comparison sites. Increasing PPFP uptake among women who deliver at home is critical to increasing contraceptive prevalence in countries like Ethiopia, where many women deliver outside facilities and do not quickly adopt contraception.^25^ Before this study, Blazer and Prata published a review in 2016 of the latest evidence on the efficacy of PPFP interventions in low- and middle-income countries.^26^ They found few recent studies on community-based programs, mainly from Asia and the Middle East using home visits; these studies had mixed results and rigor. This study fills a gap on the efficacy of community-based PPFP programs “particularly in areas with low facility-based delivery and postnatal care”^26^ as it demonstrates the benefit of integrating PPFP messages and services into existing community-level services in Ethiopia, which are provided through home visits as well as at health posts.

Contacts with HEWs at health posts and immunization outreach seem to have contributed more to the success of the intervention than home visits. While women in intervention areas received family planning information at home visits more frequently than women in comparison areas, the number was small. This finding is consistent with previous studies showing HEWs spend less time on home visits,^27,28^ plus many WDA volunteers were found to be inactive in the early months of the study.

Health centers in intervention and comparison areas received similar inputs, yet women in intervention areas more frequently received family planning information at health centers. We can speculate on reasons, including: intervention health centers performed better because of extra inputs provided during the study (two additional supervision visits plus earlier introduction of tally sheets and dashboards); the community intervention contributed to improved performance at health centers given that health posts refer to health centers; or women in intervention areas initiated conversations on PPFP at health centers after hearing about it in the community. Better performance at intervention health centers may also be due to factors we did not measure, such as strong leadership, champion providers, or client volume. In any case, health center factors may have contributed to better uptake in the intervention arm, in addition to the community-level interventions.

PPFP adoption among women delivering in facilities reached approximately 60% by 12 months postpartum in both arms. This community intervention did not increase PPFP adoption among women who delivered in facility, though it is interesting to note there was lower uptake among women in the intervention arm in the initial months postpartum, but both arms had the same cumulative adoption rate by 12 months. The community intervention, with repeat nudges before and after birth, may have helped the intervention arm “catch up” with the comparison arm, though we do not have conclusive evidence.

Nationally, mCPR is 35% among women at 12 months postpartum.^25^ Multiple recent papers have reported a wide range of mCPR among various populations of postpartum women in Ethiopia. One previous study was done in a population with a low rate of facility delivery and found mCPR was just 10.3% among women 1–12 months postpartum.^20^ Other studies that were done in populations with high facility delivery rates, living in urban areas reported a range from 38.3% mCPR in Tigray Region^21^ up to 80.3% in Addis Ababa.^29^ These studies were cross-sectional and included women at different points postpartum, so they are not directly comparable to cumulative PPFP adoption used as the outcome in this study. However, PPFP adoption among facility births in this study (60% in intervention arm, 62% in comparison arm) is similar to mCPR seen among women 10–12 months postpartum in urban Gondar in Amhara Region (57%),^22^ even though mCPR in Amhara is generally much higher than in Oromia (47% vs 28% among all married women^2^).

The absolute difference between study arms, 8.8%, is low compared to impact reported from studies of other interventions that increased PPFP use.^26,30^ However, this study looked at the additional benefit of a community component on top of strengthening PPFP at health centers. Also, the community intervention was low intensity with short trainings for HEWs (who then oriented WDA), provision of paper-based tracking tools, and quarterly supervision. In addition, many participants were in advanced stages of pregnancy when the community work started and therefore could not benefit from PPFP integration in early pregnancy, which may have dampened the effect of the intervention.

Based on the positive results from this study, Ethiopia would likely increase contraceptive use among postpartum women by directing resources toward sensitizing HEWs and WDA to the benefits of PPFP and integrating counseling and, if appropriate, provision of contraception at existing community-level contacts with pregnant women and mothers of infants. In addition, the MOH can prioritize rolling out the recently revised integrated maternal and child health card, which includes prompts for HEWs to offer integrated PPFP services based on early results from this study.^31^ While this study conducted HEW training over 4 days, some of that time was devoted to preparing HEWs to train WDA volunteers to use a tool that proved not useful^31^ and to refresher training on implant insertion, so it may be possible to reduce the duration of training while still realizing improvements in contraceptive uptake. It is also possible that alternative approaches to training HEWs to proactively counsel pregnant and postpartum women about PPFP could take the place of traditional training and be more feasible to implement at scale. The study team has already prepared an orientation package for *woreda* staff to roll out elements of this intervention during three sequential monthly meetings with PHCU heads and HEWs, taking advantage of meetings that already occur every month.

The main study strength was the pragmatic design looking at the effect of strengthening existing health services. Limitations include potential for recall bias since women may not accurately recall timing of contraception adoption or number of contacts with the health system. Health centers in both arms received inputs, so the effect of the community intervention in absence of facility strengthening is unknown. This study may have failed to capture the full effect of the intervention because data extracted from facilities show pre-discharge contraception uptake increased over time, but most women in the cohort delivered soon after implementation started. On the other hand, it is possible the effect was overestimated and was partly due to facility-level differences, not solely the community-based intervention. Facility-level differences by arm due to study implementation were described; in addition, one comparison health center only received 5 supervision visits (instead of 6) due to civil unrest. Contraceptive adoption among study participants may have been higher than otherwise seen among a similar population. Not only were contraceptive services strengthened at health centers in all study areas, but participation in enrollment interviews, which included questions on fertility desires and contraceptive options, may have increased the likelihood of adoption.

## CONCLUSIONS

This study suggests integrating PPFP into existing community-based care along the pregnancy-to-extended-postpartum continuum is promising for improving uptake in populations with high rates of home delivery. PPFP should be seen as one essential component of maternity care women receive at home (and facility) since postpartum contraceptive use can dramatically improve the health of women and their children.

## Figures and Tables

**Table 1.** Study inputs for each study arm

	Intervention	Comparison
**Zonal and *Woreda* Health Office**		
Initial meeting to explain study purpose and procedures Half-day orientation to encourage informed choice and voluntary contraceptive use Joint study supervision visits with study staff Regular meetings on findings from supervision visits Advocacy for Women Development Army revitalization	Yes	Yes
**Health Center**		
Providers of antenatal, labor & delivery, and postnatal care and immunization services trained in PPFP counseling with a contraceptive technology update in August 2016 (3 days)	Yes	Yes
Labor & delivery providers trained in postpartum IUD insertion plus refresher on implant insertion and removal in October 2016 (4 days)	Yes	Yes
Post-training follow-up visit conducted with observations of service provision to reinforce knowledge and competency	Yes	Yes
Postpartum IUD kits and supplies provided	Yes	Yes
Postpartum IUD insertion and follow-up registers introduced to record timing of insertion, information about the delivery, follow-up received, and complications	Yes	Yes
Tally sheets introduced to track the number of women counseled and PPFP provided during other maternal, newborn, and child health services; wall-mounted dashboards introduced to analyze and discuss service provision on a weekly basis	Yes (July 2017)	Yes (Nov 2017)
Primary health care unit directors received half-day orientation to encourage informed choice and voluntary contraceptive use	Yes	Yes
Supportive supervision conducted on PPFP (included: observation of counseling and services and feedback, record review, extracting service data, provider interviews at 1^st^ and last visit)	Yes (8 visits)	Yes (5-6 visits)
Replacement training on PPFP counseling and service provision provided in November 2017 (7 days)	Yes	Yes
**Health Post**		
HEWs trained on PPFP counseling with refresher on implant insertion and introduction of Nexplanon^™^ in April 2017 (4 days)	Yes	No
Modified integrated maternal and child health card (kept at health post) introduced to capture PPFP counseling and service provision and track woman’s risk of unintended pregnancy	Yes	No
Modified tally sheet introduced as support for monthly reporting to aggregate data on PPFP counseling and services	Yes	No
*Kebele* leaders received half-day orientation to encourage informed choice and voluntary contraceptive use	Yes	No
Supportive supervision on PPFP conducted	Yes (4 visits)	No
**Women’s Development Army**		
Half-day orientation by HEWs on PPFP and use of a pictorial tracking tool as job aide	Yes	No
PPFP discussed during existing monthly meetings with HEWs and supportive supervision visits	Yes	No
**Home**		
Health centers and health posts given modified tetanus toxoid cards for mothers to document her method choice and improve efficiency of counseling and service provision after birth	Yes	No

HEW: health extension workers, PPFP: postpartum family planning, IUD: intrauterine device

**Table 2.** Sociodemographic characteristics of study participants

	Intervention	Comparison	*P*-value
	N=390	N=382	
**Average age (range)**	26.5 (15-41)	26.1 (15-45)	0.189
**Married (%)**	383 (98.2)	375 (98.2)	0.969
**Religion (%)**			
Muslim	244 (62.6)	228 (59.7)	
Orthodox	139 (35.6)	152 (39.8)	0.35
Other	7 (1.8)	2 (0.5)	
**Education (%)**			
No education	170 (43.6)	183 (47.9)	
Primary	174 (44.6)	171 (44.8)	0.133
Secondary or higher	46 (11.8)	28 (7.3)	
**Household income sources**^†^ **(%)**			
Farming	356 (91.3)	370 (96.9)	0.007*
Raising livestock	50 (12.8)	129 (33.8)	<0.001*
Trade	36 (9.2)	46 (12.0)	0.290
Govt/civil servant	9 (2.3)	5 (1.3)	0.305
Other	23 (5.9)	6 (1.6)	0.017*
National wealth quintiles	N=351	N=355	
Lowest	60 (17.1%)	58 (16.4%)	
Second	49 (14.0%)	59 (16.6%)	
Middle	48 (13.7%)	89 (25.1%)	0.007*
Fourth	85 (24.2%)	84 (23.7%)	
Highest	109 (31.1%)	65 (18.4%)	

Pearson chi-squared test with Rao-Scott correction were used for categorical variables, adjusted t-test for continuous variables

† More than 1 response allowed

* *P*<0.05

**Table 3.** Number of study participants who started contraception by time of endline interview (10–16 months postpartum), method(s) used, source for method(s) used, and reasons for non-use by arm

	Intervention	Comparison	
	N=351	N=355	*P*-value
Started modern contraception by endline	48.2%	39.4%	0.061
Method^†^			
Intrauterine device	1.4%	0.0%	0.020*
Implant	9.1%	5.6%	0.101
Injectable	33.3%	29.0%	0.281
Pills	5.7%	5.1%	0.702
Female sterilization	0.0%	0.9%	0.086
Condoms	0.0%	0.0%	n/a
Lactational amenorrhea	1.7%	0.6%	0.149
Source(s) for method^†^	N=169	N=140	
Health extension worker	34.9%	34.3%	0.929
Hospital or health center	50.3%	55.0%	0.473
Private provider or drug shop	15.4%	7.9%	0.067
Other	3.6%	1.4%	0.257
Reasons for non-use^†^	N=182	N=215	
Amenorrhea	58.2%	52.1%	0.268
Pregnant	9.9%	11.6%	0.591
Side effects	8.8%	7.4%	0.642
Breastfeeding	5.5%	5.1%	0.870
Not married/having sex	7.7%	2.3%	0.011*
Husband opposed	1.7%	4.7%	0.120
Religion	1.1%	5.1%	0.038*
Inaccessible	1.1%	1.9%	0.538
Other reason	3.3%	4.7%	0.486
Unspecified	6.7%	8.8%	0.382

† More than 1 response allowed

* *P*<0.05

**Table 4.** Adoption of modern contraception over the first year postpartum by arm and place of delivery: an adjusted Cox proportional hazards model

	n	adjHR	p-value	95% CI	
				lower bound	upper bound
**Arm and place of delivery**					
Comparison arm, delivered at home	228	1.00			
Comparison arm, delivered at facility	124	2.23	<0.001*	1.54	3.25
Intervention arm, delivered at home	197	1.45	0.037*	1.02	2.04
Intervention arm, delivered at facility	151	2.24	<0.001*	1.57	3.21
**Age of woman**					
20 or younger	76	0.86	0.476	0.57	1.30
21-25	187	1.00			
26-30	278	0.82	0.123	0.64	1.05
31-35	97	0.59	0.036*	0.36	0.97
36+	62	0.52	0.013*	0.31	0.87
**Marital status**					
Not married	15				
Married	685	1.37	0.545	0.50	3.76
**Education**					
None	287	1.00			
Some primary	338	1.29	0.068	0.98	1.71
Some secondary or higher	75	1.71	0.007*	1.16	2.53
**Live children**					
0	18	1.00			
1-2	291	1.25	0.668	0.44	3.54
3-4	191	1.17	0.766	0.41	3.32
5+	200	0.78	0.650	0.26	2.34
**Religion**					
Orthodox	268	1.00			
Muslim	424	0.44	<0.001*	0.33	0.57
Other	8	1.08	0.881	0.41	2.80
**National wealth quintile**					
Lowest	118	1.00			
Second	106	0.85	0.596	0.46	1.56
Middle	135	0.89	0.629	0.55	1.44
Fourth	167	1.06	0.816	0.64	1.78
Highest	174	0.94	0.832	0.55	1.63
***Total***		*700*			

* *P*<0.05

## Data Availability

A de-identified dataset of data collected during interviews with women and a data dictionary have been submitted to the USAID Development Data Library (DDL), per donor requirements. For researchers unable to access the data from USAID, requests for the de-identified data can be made to Jhpiego (OpenDataHelp@jhpiego.org). Data sharing is subject to Jhpiego and Johns Hopkins University sharing policies and data use agreements.
